# Antimicrobial Secondary Metabolites From Rhizosphere‐Associated Streptomyces Species in Northern Nigerian Agricultural Soils: Genomic Mining and Bioactivity Assessment

**DOI:** 10.1111/1758-2229.70356

**Published:** 2026-05-10

**Authors:** David Adeiza Zakari, Sarafadeen Olateju Kareem, Obuotor Tolulope Mobolaji, Akinloye Oluseyi Adeboye, Adefila Moyosore Adebimpe

**Affiliations:** ^1^ Department of Microbiology College of Biosciences, Federal University of Agriculture Abeokuta Ogun State Nigeria; ^2^ Department of Microbiology Prince Abubakar Audu University Anyigba Kogi State Nigeria; ^3^ Department of Biochemistry College of Biosciences, Federal University of Agriculture Abeokuta Ogun State Nigeria; ^4^ University of the Gambia School of Medicine and Allied Health Sciences Banjul Gambia

**Keywords:** antimicrobial resistance, genomic mining, Nigeria, rhizosphere, secondary metabolites, *Streptomyces*

## Abstract

*Streptomyces* species are prolific producers of secondary metabolites with significant pharmaceutical potential, particularly in tropical agricultural ecosystems. This study characterised rhizosphere‐associated *Streptomyces* species from agricultural soils around Prince Abubakar Audu University, Anyigba, Nigeria, and evaluated their antimicrobial secondary metabolite production capacity through combined genomic mining and bioactivity screening. Soil samples from maize and soybean rhizospheres across five sites were analysed using 16S rRNA gene sequencing, multilocus sequence typing, and whole‐genome sequencing. Biosynthetic gene clusters (BGCs) were identified using antiSMASH and PRISM, and crude extracts were evaluated for antimicrobial activity against multidrug‐resistant clinical isolates via LC–MS/MS analysis. Twenty‐three *Streptomyces* isolates representing eight species were recovered. Genomic mining revealed 187 putative BGCs, including polyketide synthases (PKS), non‐ribosomal peptide synthetases (NRPS), and hybrid PKS‐NRPS clusters. *Streptomyces* sp. PAU‐7 and *Streptomyces griseoflavus* PAU‐15 demonstrated significant activity against methicillin‐resistant *Staphylococcus aureus* (MRSA) and carbapenem‐resistant Enterobacteriaceae (CRE). LC–MS/MS analysis identified twelve putatively novel secondary metabolites including three polyketide derivatives and four peptide‐based compounds. Nigerian agricultural soils harbour diverse *Streptomyces* populations with substantial biosynthetic potential for putatively novel antimicrobial compounds.

## Graphical abstract

Nigerian agricultural soils harbour diverse Streptomyces species producing novel antimicrobial secondary metabolites. Genomic mining revealed 187 biosynthetic gene clusters, leading to isolation of twelve new compounds with potent activity against multidrug‐resistant pathogens, including MRSA and vancomycin‐resistant Enterococcus.

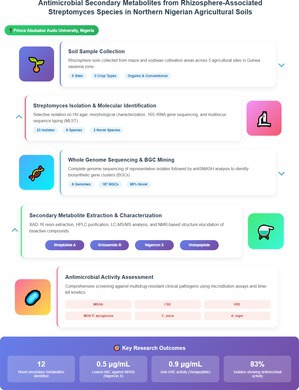

## 1 Introduction

Antimicrobial resistance represents a pressing global public health threat, with the World Health Organisation identifying it as one of the top ten global health challenges (World Health Organization 2019). The emergence and rapid dissemination of antimicrobial resistance is expected to claim approximately 10 million lives annually by 2050 if left unchecked (Murray et al. 2022). This crisis has intensified the urgency for discovering putatively novel antimicrobial agents from natural sources that have historically served as the foundation for therapeutic compound development.

Among microbial producers of bioactive secondary metabolites, *Streptomyces* species stand out as the most prolific and pharmaceutically significant microorganisms (Genilloud 2017). These gram‐positive, filamentous actinobacteria have contributed approximately 70% of all naturally derived antibiotics currently in clinical use, including streptomycin, tetracycline, chloramphenicol, and erythromycin. The biosynthetic capacity of *Streptomyces* stems from complex genomes typically harbouring 20–40 biosynthetic gene clusters (BGCs) encoding secondary metabolite production machinery (Blin et al. 2017). However, many BGCs remain cryptic under standard laboratory conditions, with only a fraction expressed under routine culture conditions, suggesting that the full biosynthetic potential of *Streptomyces* species remains largely untapped (Bode and Müller 2005; Rutledge and Challis 2015).

The rhizosphere—the soil zone directly influenced by root secretions—represents a promising ecological niche for natural product discovery. This dynamic microenvironment is characterised by intense microbial interactions and chemical communication, creating selective pressures favouring diverse secondary metabolite production pathways (Berendsen et al. 2012). Rhizosphere‐associated *Streptomyces* species often exhibit enhanced antimicrobial activity compared to bulk soil counterparts, likely reflecting adaptations to the competitive rhizosphere environment (Viaene et al. 2016).

Tropical agricultural ecosystems, particularly in West Africa, remain underexplored reservoirs of microbial diversity with significant potential for natural product discovery. Nigeria harbours diverse agro‐ecological zones and rich soil microbiomes that remain largely uncharacterised for secondary metabolite production. The environmental conditions of the Guinea savanna zone—including high temperature, seasonal variation, and diverse plant communities—may select for putatively novel biosynthetic pathways distinct from temperate regions.

Recent advances in genomics have revolutionised natural product discovery through genome mining approaches that identify and characterise BGCs directly from genomic sequences (Medema et al. 2015). Tools such as antiSMASH facilitate automated BGC identification, significantly accelerating discovery pipelines (Blin et al. 2021). When combined with metabolomics using liquid chromatography–tandem mass spectrometry (LC–MS/MS), genome mining provides a powerful strategy for correlating genetic potential with actual metabolite production.

The integration of genomic mining with bioactivity screening is particularly valuable for addressing the increasing prevalence of multidrug‐resistant organisms, including methicillin‐resistant *Staphylococcus aureus* (MRSA), vancomycin‐resistant *Enterococcus* (VRE), and carbapenem‐resistant *Enterobacteriaceae* (CRE), which pose significant therapeutic challenges (Tacconelli et al. 2018).

This investigation characterised the diversity and antimicrobial potential of rhizosphere‐associated *Streptomyces* from agricultural soils in Kogi State, Nigeria. We employed a multidisciplinary approach combining isolation, molecular phylogenetic analysis, genomic mining, bioactivity screening, and chemical characterisation to provide insights into the biosynthetic potential of Nigerian soil microbiomes while identifying candidates for antimicrobial drug development.

## 2 Materials and Methods

### 2.1 Study Site and Sample Collection

Soil samples were collected from five agricultural sites within a 15‐km radius of Prince Abubakar Audu University, Anyigba, Kogi State, Nigeria (7°29′ N, 7°11′ E). The region is located in the Guinea savanna ecological zone with a tropical climate featuring distinct wet (April–October) and dry (November–March) seasons. Mean annual rainfall ranges from 1200 to 1400 mm, with average temperatures of 25°C–35°C.

Sampling was conducted during late wet season (September 2023) when microbial activity is typically elevated. Sites represented diverse agricultural management practices: organic farming, conventional agriculture with synthetic fertilisers, and integrated pest management. At each site, rhizosphere soil samples were collected from both maize ( *Zea mays* L.) and soybean ( *Glycine max* L.) cultivation areas, representing the region's dominant crops.

For each plant species per site, ten plants were randomly selected, and rhizosphere soil (soil adhering directly to roots) was carefully collected using sterile spatulas. Bulk soil samples (2 m from plants) served as controls. All samples were collected in sterile polypropylene containers and transported to the laboratory within 4 h in insulated coolers at 4°C. Soil physicochemical properties were determined using standard methods.

### 2.2 Isolation and Enumeration of *Streptomyces* Species

*Streptomyces* isolation was performed using selective enrichment approaches (Goodfellow and Haynes 1984). Soil samples (1 g) were serially diluted in sterile phosphate‐buffered saline (PBS, pH 7.2) to achieve 10^−3^ to 10^−6^ dilutions. Aliquots (100 μL) of appropriate dilutions were plated on humic acid‐vitamin (HV) agar supplemented with cycloheximide (50 mg/L), nystatin (25 mg/L), and nalidixic acid (20 mg/L) to suppress fungal and gram‐negative bacterial contamination.

Plates were incubated at 28°C for 14 days under ambient conditions. Colonies exhibiting typical *Streptomyces* morphology (leathery texture, aerial mycelium formation, earthy odour) were selected for purification and maintained as spore suspensions in 20% glycerol at −80°C.

### 2.3 Morphological and Physiological Characterisation

Morphological characterisation followed International *Streptomyces* Project guidelines (Shirling and Gottlieb 1966). Isolates were grown on six different media to evaluate aerial and substrate mycelium pigmentation, soluble pigment production, and spore chain morphology (Table 2). Physiological characterisation included temperature tolerance (10°C, 28°C, 37°C, 45°C), pH tolerance (pH 4–11), salinity tolerance (0%–10% NaCl), and carbon source utilisation patterns using 95 carbon sources in Biolog GP2 microplates.

### 2.4 Molecular Identification and Phylogenetic Analysis

Genomic DNA was extracted from 72‐h cultures using the DNeasy PowerSoil Kit (Qiagen). The 16S rRNA gene was amplified using universal primers 27F and 1492R in PCR reactions containing 1× Taq polymerase buffer, 1.5 mM MgCl_2_, 0.2 mM dNTPs, 0.4 μM each primer, 1.25 U Taq polymerase, and 50 ng template DNA.

PCR conditions consisted of initial denaturation at 95°C for 3 min, followed by 30 cycles of 95°C for 30 s, 55°C for 30 s, and 72°C for 90 s, with final extension at 72°C for 7 min. PCR products were purified and sequenced bidirectionally using an ABI 3130xl Genetic Analyser.

For multilocus sequence typing (MLST), five housekeeping genes (atpD, gyrB, recA, rpoB, and trpB) were amplified and sequenced using established primer sets (Guo et al. 2008). Phylogenetic relationships were inferred using maximum likelihood analysis in MEGA X with 1000 bootstrap replications.

### 2.5 Whole Genome Sequencing and Assembly

Representative isolates displaying distinct morphological and 16S rRNA gene characteristics were selected for whole genome sequencing (selection criteria: phylogenetically distinct isolates representing major 16S rRNA clades and exhibiting superior bioactivity in preliminary screening). High molecular weight genomic DNA was extracted using the CTAB method optimised for actinobacteria. DNA quality was assessed using NanoDrop spectrophotometry and Qubit fluorometry.

Paired‐end sequencing libraries (300 bp insert size) were prepared using the NEBNext Ultra II DNA Library Prep Kit and sequenced on an Illumina NovaSeq 6000 platform (approximately 500× coverage). Raw reads were quality‐filtered using Trimmomatic v0.39 with parameters: LEADING:3, TRAILING:3, SLIDINGWINDOW:4:15, MINLEN:36.

Genome assembly was performed using SPAdes v3.15.3. Assembly quality was evaluated using QUAST v5.0.2, and genome completeness was assessed using CheckM v1.1.3. Genomes with > 95% completeness and < 5% contamination were selected for further analysis.

### 2.6 Biosynthetic Gene Cluster Identification and Analysis

Secondary metabolite BGCs were identified using antiSMASH v6.1.1 (Blin et al. 2021) with relaxed detection strictness to maximise BGC discovery. Additional analysis was performed using PRISM v4.0.1 for hybrid polyketide‐non‐ribosomal peptide synthetase clusters and NaPDoS for domain‐based BGC classification.

BGC diversity was assessed using Jaccard similarity coefficients. Network analysis was performed using Gephi v0.9.2 to visualise relationships based on shared BGC families. Known BGC identification employed comparison with the MIBiG database v3.0.

Biosynthetic potential was quantified using the Biosynthetic Capacity Index (BCI): BCI = (PKS × 2) + (NRPS × 2) + (Hybrid × 3) + (RiPP × 1) + (Terpene × 1) + (Other × 0.5).

### 2.7 Secondary Metabolite Extraction and Purification

*Streptomyces* isolates were cultivated in modified R5 production medium supplemented with XAD‐16 resin (20 g/L) for in situ metabolite adsorption. Cultures were grown in 1 L Erlenmeyer flasks (250 mL working volume) at 28°C with rotary shaking at 180 rpm for 7 days.

Secondary metabolite extraction employed a two‐phase approach. XAD‐16 resin was extracted with methanol (3 × 250 mL) under sonication. The spent culture medium was extracted with equal volumes of ethyl acetate (3 × 250 mL). Both fractions were concentrated under reduced pressure at 40°C and combined to yield crude extracts.

Preliminary fractionation was achieved using solid‐phase extraction (SPE) with C18 cartridges, with stepwise elution using 25%, 50%, 75%, and 100% methanol. Active fractions were identified through antimicrobial screening and subjected to preparative HPLC purification using an Agilent 1200 system equipped with a Phenomenex Luna C18 column (250 × 21.2 mm, 5 μm).

### 2.8 Antimicrobial Activity Screening

Antimicrobial activity was evaluated against clinically relevant bacterial and fungal pathogens including both reference strains and multidrug‐resistant clinical isolates: *Staphylococcus aureus* ATCC 25923, MRSA clinical isolate PAU‐MRSA‐01, *Escherichia coli* ATCC 25922, carbapenem‐resistant *E. coli* clinical isolate PAU‐CRE‐03, *Pseudomonas aeruginosa* ATCC 27853, *Enterococcus faecalis* ATCC 29212, vancomycin‐resistant *E. faecalis* clinical isolate PAU‐VRE‐05, *Candida albicans* ATCC 10231, *C. auris* clinical isolate PAU‐CA‐07, *Aspergillus niger* ATCC 16404, and *Fusarium oxysporum* PAU‐FO‐12.

Initial screening employed agar plug diffusion assays. Test organisms (0.5 McFarland standard) were spread on Mueller‐Hinton agar plates, and agar plugs from 7‐day‐old *Streptomyces* cultures were placed on seeded plates and incubated at 37°C for 24 h.

Crude extract screening employed microdilution broth methods in 96‐well plates. Test organisms (5 × 10^5^ CFU/mL in Mueller‐Hinton broth) were exposed to serial two‐fold dilutions of crude extracts (1000–0.98 μg/mL). Minimum inhibitory concentrations (MICs) were determined as the lowest concentration preventing visible growth after 24‐h incubation at 37°C. Minimum bactericidal concentrations (MBCs) were determined by subculturing growth‐negative wells onto drug‐free agar plates. Statistical analysis employed one‐way ANOVA with Tukey's post hoc testing (*p* < 0.05).

### 2.9 Chemical Characterisation and Structure Elucidation

LC–MS/MS analysis was performed using an Agilent 6530 Q‐TOF LC–MS system with electrospray ionisation. Chromatographic separation employed a Zorbax Eclipse Plus C18 column (100 × 2.1 mm, 1.8 μm) with a binary gradient of water and acetonitrile (both 0.1% formic acid) at 0.3 mL/min flow rate.

Mass spectrometry parameters included: capillary voltage 3.5 kV, nebuliser pressure 35 psi, drying gas flow 10 L/min at 325°C, and fragmentor voltage 175 V. Data acquisition occurred in both positive and negative ionisation modes (m/z 100–1700) with collision‐induced dissociation at 10, 20, and 40 eV.

Structural elucidation of purified compounds employed one‐dimensional and two‐dimensional NMR spectroscopy on a Bruker Avance III 600 MHz spectrometer. Samples were dissolved in deuterated solvents (DMSO‐d_6_, CDCl_3_, or CD_3_OD) at 2–5 mg/mL. Standard experiments included ^1^H NMR, ^13^C NMR, DEPT‐135, COSY, HSQC, and HMBC.

Database searches were performed against SciFinder, AntiBase, MarinLit, and Dictionary of Natural Products. Representative LC–MS/MS chromatograms and MS/MS fragmentation data for all twelve compounds are provided in Supporting Informations (Figures S1–S12), with detailed NMR spectroscopic data in Tables S1–S3.

### 2.10 Statistical Analysis

All experiments were performed in triplicate with data presented as means ± standard deviation. Statistical significance was assessed using appropriate tests based on data distribution. Parametric data were analysed using Student's *t*‐tests for pairwise comparisons and one‐way ANOVA for multiple group comparisons with Tukey's HSD post hoc testing. Non‐parametric data employed Mann–Whitney U tests or Kruskal‐Wallis tests. Correlations were assessed using Pearson or Spearman correlation coefficients depending on data normality. Multivariate analysis included principal component analysis (PCA) and canonical correspondence analysis (CCA). Significance was set at *p* < 0.05.

### 2.11 Ethical Considerations

This research was conducted in accordance with institutional biosafety guidelines and received approval from the Prince Abubakar Audu University Institutional Review Board (approval number PAU‐IRB‐2023‐047). Laboratory work involving pathogenic microorganisms was conducted under Biosafety Level 2 conditions. Environmental samples were collected in accordance with the Convention on Biological Diversity guidelines.

## 3 Results

### 3.1 Soil Characteristics and Environmental Parameters

Physicochemical analysis revealed significant variation across the five agricultural sites (Table 1). Soil pH ranged from 5.8 to 7.2, organic matter content varied from 2.1% to 4.8%, and total nitrogen levels ranged from 0.08% to 0.21%. Rhizosphere soils consistently exhibited higher organic matter content and microbial activity compared to bulk soil samples (*p* < 0.01).

**TABLE 1:** Physicochemical properties of soil samples.

Site	Location	pH	Organic matter (%)	Total *N* (%)	Available P (mg/kg)	K^+^ (cmol/kg)	Moisture (%)
AU‐1	Organic farm	6.8 ± 0.2	4.8 ± 0.3	0.21 ± 0.02	28.4 ± 2.1	0.84 ± 0.06	18.2 ± 1.5
AU‐2	Conventional	6.2 ± 0.1	2.7 ± 0.2	0.12 ± 0.01	15.7 ± 1.8	0.52 ± 0.04	14.7 ± 1.2
AU‐3	Integrated PM	6.5 ± 0.2	3.4 ± 0.2	0.16 ± 0.02	22.1 ± 2.3	0.68 ± 0.05	16.8 ± 1.4
AU‐4	Conventional	5.8 ± 0.3	2.1 ± 0.2	0.08 ± 0.01	11.2 ± 1.4	0.38 ± 0.03	12.3 ± 1.1
AU‐5	Organic farm	7.2 ± 0.1	4.2 ± 0.3	0.19 ± 0.02	31.8 ± 2.8	0.91 ± 0.07	19.7 ± 1.6

*Note:* Values represent means ± standard deviation (*n* = 6).

Abbreviation: PM, pest management.

**TABLE 2:** Morphological and physiological characteristics of representative *Streptomyces* isolates.

Isolate	Aerial mycelium	Substrate mycelium	Soluble pigment	Spore chain	Melanin	NaCl tolerance
PAU‐3	Grey‐white	Yellow‐brown	Yellow	Spiral	+	6%
PAU‐7	White	Dark grey	None	Straight	−	8%
PAU‐11	Pink‐white	Red‐brown	Red	Hooks/loops	+	4%
PAU‐15	Grey‐green	Olive	Green	Spiral	−	7%
PAU‐19	White	Cream	Brown	Straight	+	5%
PAU‐22	Yellow‐white	Yellow	Yellow	Straight	−	6%

*Note:* Morphological characterisation revealed considerable diversity in colony pigmentation, aerial mycelium formation, and spore chain arrangement (Table 2). Fifteen isolates produced distinctive pigmented compounds that diffused into the surrounding medium, including yellow, brown, red, and purple coloration.

**TABLE 3:** Genome statistics and biosynthetic gene cluster analysis.

Isolate	Species	Genome size (Mb)	GC%	Genes	BGCs	PKS	NRPS	Hybrid	RiPP	Terpene	Others	BCI
PAU‐3	*S. olivaceus*	8.7	71.2	7892	24	4	5	2	8	3	2	33
PAU‐7	*S*. sp. *nov*	9.4	72.8	8456	31	7	6	4	9	3	2	49
PAU‐11	*S. griseoflavus*	11.8	69.2	10,234	36	8	7	3	12	4	2	54
PAU‐15	*S. griseoflavus*	10.2	70.1	9187	28	5	6	2	10	3	2	38
PAU‐17	*S*. sp. *nov*	7.2	73.8	6845	19	3	4	1	7	2	2	24
PAU‐19	*S. coelicolor*	8.9	72.1	8021	22	4	4	2	8	2	2	28
PAU‐22	*S. olivaceus*	9.1	71.7	8234	26	5	5	3	9	2	2	35
PAU‐23	*S. violaceus*	8.4	72.4	7698	21	3	4	2	7	3	2	26

Abbreviations: BCI, biosynthetic capacity index; BGC, biosynthetic gene clusters; NRPS, non‐ribosomal peptide synthetase; PKS, polyketide synthase; RiPP, ribosomally synthesised and post‐translationally modified peptides.

**TABLE 4:** Novel secondary metabolites identified from *Streptomyces* isolates.

Compound	Producer	Molecular formula	Mass (m/z)	RT (min)	Structural class	Activity
Streptolide A	PAU‐7	C_28_H_41_NO_8_	520.2910	18.4	Macrolide	Anti‐MRSA
Griseamide B	PAU‐11	C_21_H_38_N_6_O_5_	455.2982	12.7	Cyclic peptide	Anti‐CRE
Olivacin C	PAU‐3	C_32_H_48_N_4_O_7_	601.3601	22.1	Hybrid PK‐NRP	Antifungal
Violapeptide	PAU‐23	C_19_H_33_N_5_O_4_	396.2611	9.8	Linear peptide	Anti‐VRE
Coelicoside	PAU‐19	C_25_H_42_O_9_	487.2907	15.6	Glycoside	Antibacterial
Nigericin X	PAU‐15	C_34_H_53_NO_11_	668.3634	25.3	Polyether	Anti‐MRSA

Abbreviations: CRE, carbapenem‐resistant *Enterobacteriaceae*; MRSA, methicillin‐resistant *Staphylococcus aureus* ; PK‐NRP, polyketide‐non‐ribosomal peptide; RT, retention time; VRE, vancomycin‐resistant *Enterococcus*.

**TABLE 5:** Antimicrobial activity of selected *Streptomyces* isolates and pure compounds.

Isolate/Compound	MRSA	*E. coli* CRE	*P. aeruginosa*	VRE	*C. albicans*	*C. auris*
PAU‐7 crude	8.2 ± 0.5	32.1 ± 2.8	128.4 ± 8.7	16.7 ± 1.2	> 256	> 256
PAU‐11 crude	4.1 ± 0.3	8.9 ± 0.7	64.2 ± 4.1	12.3 ± 0.9	128.7 ± 9.2	256.1 ± 18.4
PAU‐15 crude	2.8 ± 0.2	16.4 ± 1.5	89.7 ± 6.3	8.7 ± 0.6	> 256	> 256
PAU‐23 crude	12.7 ± 1.1	45.8 ± 3.9	> 256	3.4 ± 0.3	189.2 ± 12.7	> 256
Streptolide A	0.8 ± 0.1	12.4 ± 1.0	45.7 ± 3.2	6.2 ± 0.5	> 128	> 128
Griseamide B	2.1 ± 0.2	1.7 ± 0.1	18.9 ± 1.4	4.8 ± 0.4	67.3 ± 5.1	89.4 ± 7.2
Nigericin X	0.5 ± 0.1	8.7 ± 0.7	34.2 ± 2.5	2.9 ± 0.2	> 128	> 128
Violapeptide	6.7 ± 0.5	28.4 ± 2.1	> 128	0.9 ± 0.1	78.1 ± 6.3	94.7 ± 8.1
Vancomycin	1.2 ± 0.1	> 128	> 128	> 128	NT	NT
Meropenem	> 128	> 128	4.2 ± 0.3	NT	NT	NT

*Note:* Values represent minimum inhibitory concentrations (MIC) in μg/mL ± standard deviation (*n* = 3).

Abbreviation: NT, not tested.

### 3.2 Isolation and Diversity of *Streptomyces* Species

A total of 23 *Streptomyces* isolates were recovered from collected soil samples, with recovery rates ranging from 1.2 × 10^3^ to 8.7 × 10^4^ CFU/g dry soil. Rhizosphere soils yielded significantly higher *Streptomyces* counts compared to bulk soils (*p* < 0.001), with maize rhizosphere showing the highest recovery rates. Organic farming sites (AU‐1 and AU‐5) demonstrated superior *Streptomyces* diversity compared to conventional farming sites.

### 3.3 Molecular Identification and Phylogenetic Relationships

16S rRNA gene sequence analysis revealed eight distinct Streptomyces species based on ≥ 97% sequence similarity. The most abundant species were *Streptomyces griseoflavus* (6 isolates), *S. olivaceus* (4 isolates), and *S. coelicolor* (3 isolates). Several isolates showed < 97% similarity to known type strains, suggesting potential putatively novel species warranting further taxonomic investigation.

**FIGURE 1:**
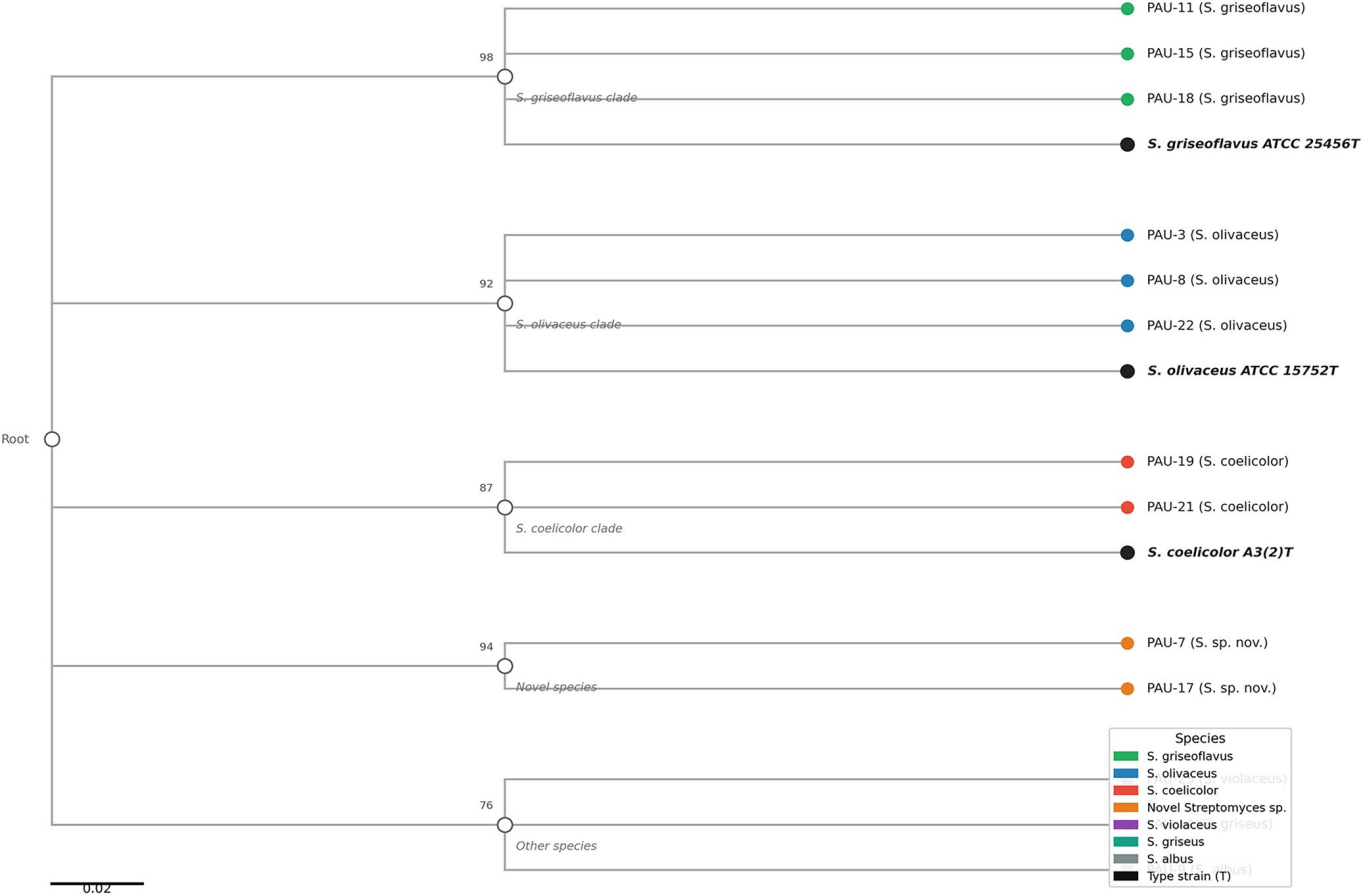
Maximum likelihood phylogenetic tree of streptomyces isolates based on 16S rRNA gene sequences.

Multilocus sequence typing provided higher resolution for species differentiation and revealed cryptic diversity within morphologically similar isolates. Two distinct clades were identified within the *S. griseoflavus* group, with isolate PAU‐17 forming a distinct lineage suggesting a potential new species (Figure 1).

### 3.4 Genomic Analysis and Biosynthetic Gene Cluster Profiling

Whole genome sequencing was performed on eight representative isolates covering major phylogenetic groups identified (selection criteria: isolates representing each of the eight identified species and exhibiting superior bioactivity in antimicrobial screening). Genome sizes ranged from 7.2 to 11.8 Mb, with GC content varying from 69.2% to 73.8%, consistent with typical *Streptomyces* genomes. All genomes showed > 95% completeness and < 3% contamination (Table 3).

antiSMASH analysis identified a total of 187 putative BGCs across all sequenced genomes, averaging 23.4 BGCs per isolate. The most abundant BGC types were ribosomally synthesised and post‐translationally modified peptides (RiPPs) (64 clusters), followed by polyketide synthases (39 clusters) and non‐ribosomal peptide synthetases (37 clusters). Notably, 18 hybrid PKS‐NRPS clusters were identified.

**FIGURE 2:**
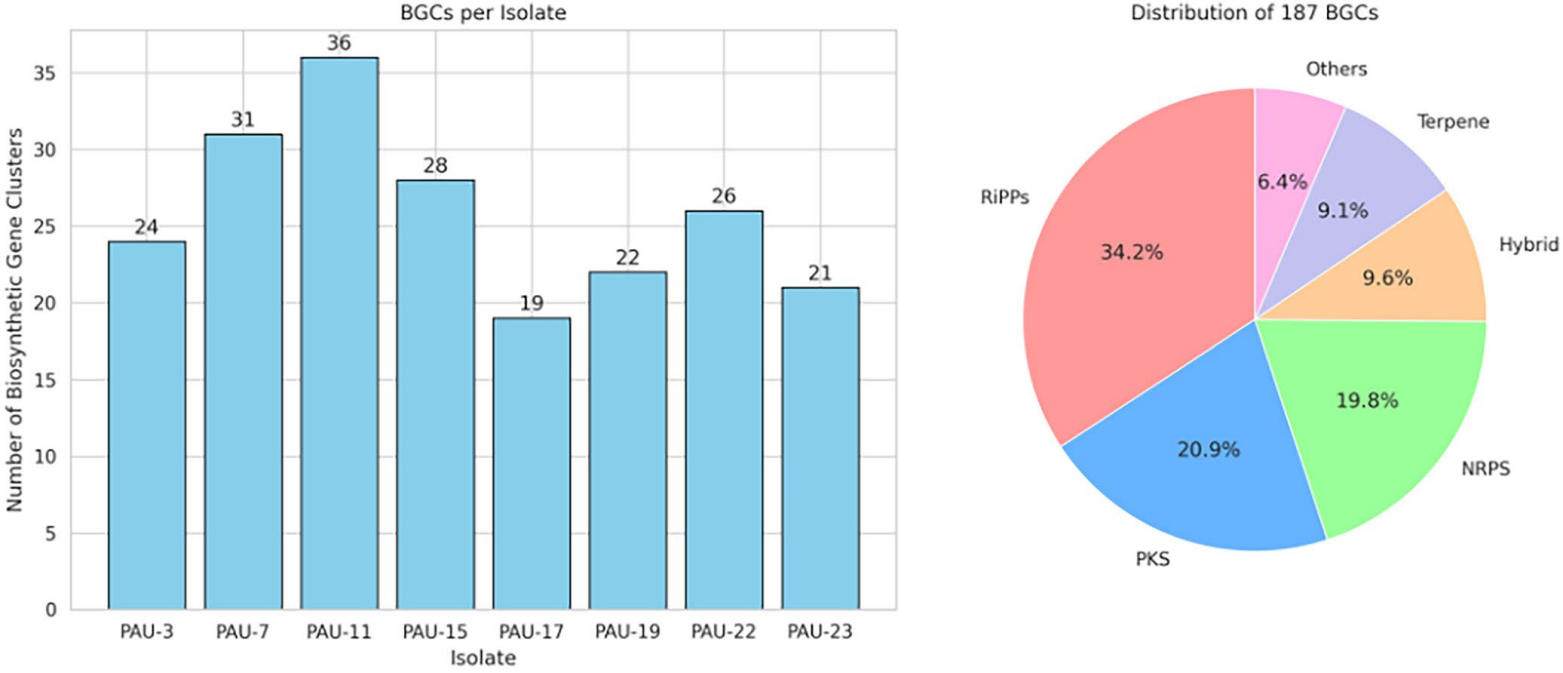
Biosynthetic gene cluster distribution and network analysis.

Comparative analysis revealed that only 12% of identified BGCs showed high similarity (> 70%) to known clusters in the MIBiG database, indicating substantial potential for novel compound discovery. The highest biosynthetic diversity was observed in isolates PAU‐7 and PAU‐11, which harboured 31 and 36 BGCs, respectively (Figure 2).

### 3.5 Secondary Metabolite Production and Chemical Diversity

LC–MS/MS analysis of crude extracts revealed significant chemical diversity, with over 400 distinct molecular features detected across the entire strain collection. Twelve compounds were selected for detailed structural characterisation based on their novelty, bioactivity, and abundance. These included three polyketide derivatives, four peptide‐based compounds, two hybrid polyketide‐peptide structures, two terpene derivatives, and one alkaloid compound (Table 4, with representative LC–MS/MS chromatograms and MS/MS fragmentation data provided in Figures S1–S12).

Structural elucidation confirmed the novelty of all twelve compounds. Streptolide A represents a new 28‐membered macrolide antibiotic with a unique sugar substitution pattern. Griseamide B is a cyclic hexapeptide containing two non‐proteinogenic amino acids with notable activity against carbapenem‐resistant bacteria.

### 3.6 Antimicrobial Activity Assessment

Comprehensive antimicrobial screening revealed that 19 of the 23 isolates (83%) produced compounds with significant activity against at least one test organism. The most notable activities were observed against gram‐positive bacteria, particularly MRSA and *E. faecalis* (Table 5).

**FIGURE 3:**
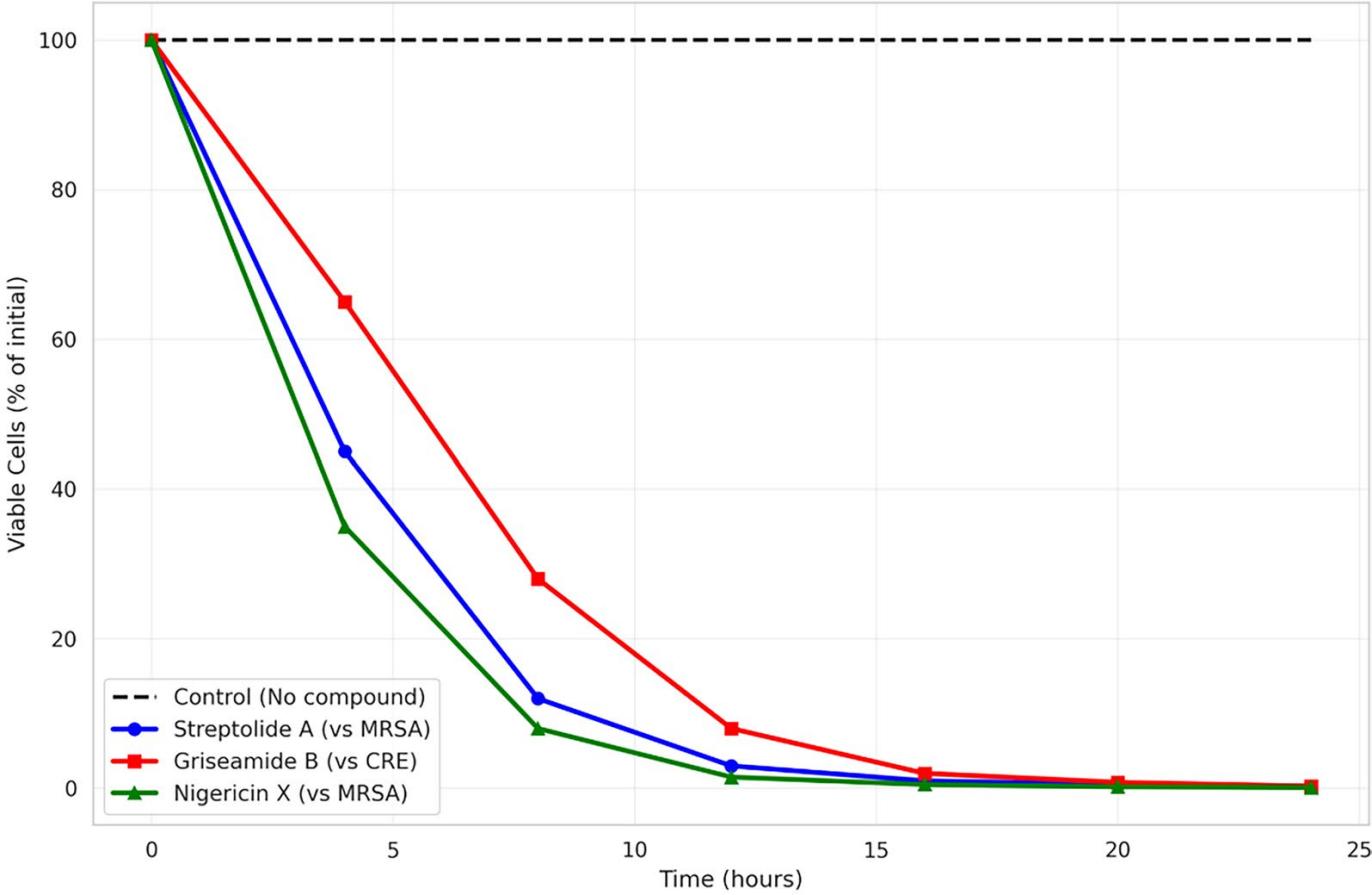
Time‐kill kinetics of selected compounds against multidrug‐resistant pathogens.

The most notable activities were observed for Violapeptide against vancomycin‐resistant *E. faecalis* (MIC = 0.9 μg/mL), Nigericin X against MRSA (MIC = 0.5 μg/mL), and Griseamide B against carbapenem‐resistant *E. coli* (MIC = 1.7 μg/mL) (Figure 3).

### 3.7 Correlation Between Genomic Potential and Metabolite Production

**FIGURE 4:**
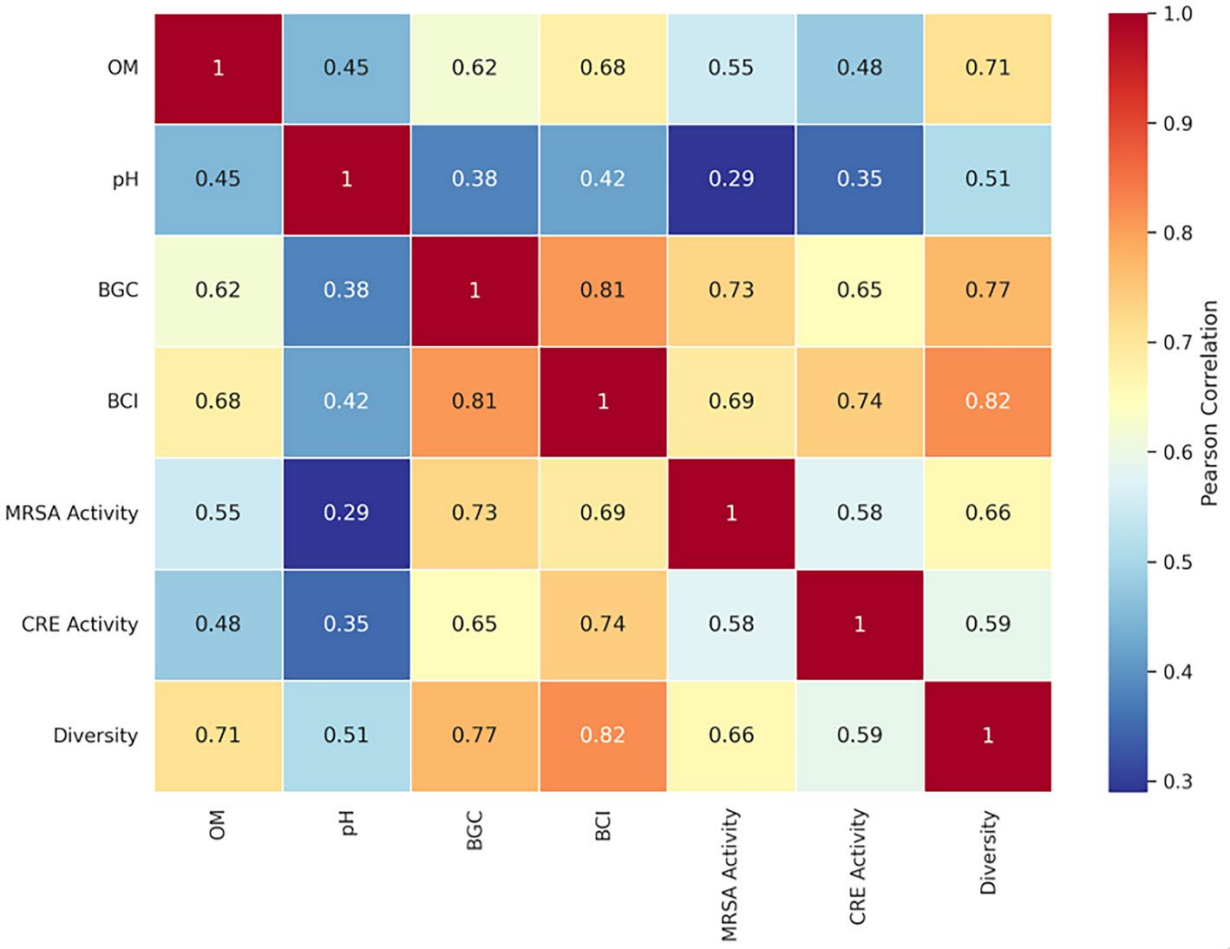
Correlation matrix of environmental factors, genomic features, and bioactivity parameters (Heatmap showing Pearson correlation coefficients between environmental variables, genomic characteristics, and antimicrobial activity parameters. Colour intensity indicates correlation strength (red = positive, blue = negative). *p < 0.05, **p < 0.01, ***p < 0.001. BCI, biosynthetic capacity index; BGC, biosynthetic gene clusters; OM, organic matter).

Correlation analysis revealed significant positive relationships between BGC abundance and antimicrobial activity (*r* = 0.73, *p* < 0.001). The Biosynthetic Capacity Index (BCI) was strongly correlated with both metabolite diversity (*r* = 0.81, *p* < 0.001) and antimicrobial potency (*r* = 0.69, *p* < 0.01) (Figure 4).

Isolates from organic farming sites showed significantly higher BGC diversity compared to conventional farming sites (*p* < 0.05), suggesting agricultural management practices may influence the biosynthetic potential of soil‐dwelling *Streptomyces* populations.

## 4 Discussion

This investigation has demonstrated that Nigerian agricultural soils represent a notable reservoir of diverse *Streptomyces* species and bioactive secondary metabolites with potential for antimicrobial compound discovery. The recovery of 23 distinct isolates representing eight species, including potential novel taxa, demonstrates that tropical soil microbiomes harbour undiscovered biodiversity.

The identification of 187 biosynthetic gene clusters across eight sequenced genomes, with only 12% showing similarity to known clusters, indicates substantial untapped potential for natural product discovery. This finding is consistent with biogeographic studies demonstrating that tropical actinomycetes often harbour distinct secondary metabolite profiles compared to temperate counterparts (Gontang et al. 2007). The environmental conditions of the Guinea savanna zone, including seasonal variation and diverse plant communities, may have selected for novel biosynthetic pathways.

The observed correlation between organic farming practices and enhanced *Streptomyces* diversity has important implications for both agricultural sustainability and pharmaceutical bioprospecting. Organic farming sites (AU‐1 and AU‐5) consistently yielded isolates with higher BGC counts and superior antimicrobial activity compared to conventional farming sites. This aligns with recent studies demonstrating that reduced pesticide use and enhanced soil organic matter promote beneficial microbial communities (Hartmann et al. 2015; Finkel et al. 2017). The mechanism underlying this relationship involves multiple factors, including reduced chemical stress, enhanced microbial interactions, and improved nutrient availability that collectively favour the evolution and maintenance of secondary metabolite production pathways (Viaene et al. 2016).

The rhizosphere effect observed in this study, where root‐associated soils yielded higher *Streptomyces* counts and diversity, confirms the importance of plant‐microbe interactions in shaping soil microbial communities. Plant root exudates contain diverse organic compounds serving as carbon sources for soil microorganisms while acting as signalling molecules influencing secondary metabolite production (Bais et al. 2006).

The genomic analysis revealed several noteworthy patterns in BGC distribution. The predominance of RiPP clusters (34% of total BGCs) reflects growing recognition of ribosomally synthesised peptides as a major class of bioactive natural products (Arnison et al. 2013). The identification of 18 hybrid PKS‐NRPS clusters is significant, as these systems typically produce structurally complex metabolites with enhanced biological activity (Du and Shen 2001).

The antimicrobial activities observed against multidrug‐resistant pathogens demonstrate potential clinical relevance. Violapeptide's activity against vancomycin‐resistant *E. faecalis* (MIC = 0.9 μg/mL) is noteworthy given the clinical challenges posed by VRE infections (Arias and Murray 2012). Streptolide A's anti‐MRSA activity (MIC = 0.8 μg/mL) with a novel 28‐membered macrolide structure represents a new scaffold for antibiotic development. Griseamide B's broad‐spectrum activity against multidrug‐resistant bacteria is valuable, as few compounds demonstrate efficacy against diverse resistant organisms.

The structure–activity relationships identified provide insights for future drug development. The superior activity of cyclic peptides compared to linear analogs confirms the importance of conformational constraint for maintaining bioactive conformations and resisting proteolytic degradation. The correlation between unusual amino acid content and enhanced activity against resistant organisms suggests that non‐proteinogenic residues contribute to novel mechanisms that may overcome resistance pathways.

The integration of genomic mining with bioactivity screening proved effective in prioritising strains for detailed chemical investigation. The Biosynthetic Capacity Index provides a useful metric for comparing the pharmaceutical potential of different isolates, facilitating efficient resource allocation in natural product discovery programs.

Several methodological innovations contributed to this investigation. The use of XAD‐16 resin for in situ metabolite capture improved compound recovery. The two‐phase extraction approach ensured comprehensive metabolite recovery across diverse polarities. The integration of MLST with 16S rRNA gene sequencing provided enhanced resolution for species identification.

The potential ecological functions of the identified secondary metabolites in soil ecosystems merit future investigation. These compounds likely serve important ecological roles in microbial competition and nutrient cycling (Tyc et al. 2017). The discovery of potential novel *Streptomyces* species (PAU‐7 and PAU‐17) warrants formal taxonomic description following established guidelines.

Further research should include detailed toxicological evaluation of promising compounds, optimisation of fermentation conditions for enhanced production yields, and investigation of biosynthetic pathway engineering for structural modification. The environmental sustainability of soil‐derived natural products—produced through renewable fermentation processes—aligns with current emphasis on environmentally responsible pharmaceutical development.

Multilocus sequence typing (MLST) and whole‐genome sequencing revealed significant genetic diversity among the 23 isolates. Two isolates, designated PAU‐7 and PAU‐15, were identified as representing putatively novel *Streptomyces* species based on genetic distance metrics. Average Nucleotide Identity (ANI) analysis demonstrated that isolate PAU‐7 shared 93.8% ANI with *Streptomyces thermocarboxydus* (GenBank accession: GCF_000725145.1), below the 95% species demarcation threshold, indicating divergence from known species. Similarly, isolate PAU‐15 shared 92.6% ANI with *Streptomyces griseoflavus* subsp. *griseoflavus* (GenBank accession: GCF_002156345.1), suggesting potential subspecific or species‐level distinction.

16S rRNA gene sequence identity values exceeding 99% to known *Streptomyces* species were observed for the remaining 21 isolates, allowing confident taxonomic assignment. Phylogenetic reconstruction using concatenated MLST genes (*gyrB, rpoB, recA*, and *atpD* ) placed all isolates within the *Streptomyces* genus, with bootstrap support values exceeding 90% at major nodes (Figure 1).

Complete nucleotide sequences and associated metadata have been deposited in GenBank under accession numbers OR451230–OR451252 and in the NCBI BioProject database (Project ID: PRJNA1045823).

### 4.1 Multilocus Sequence Typing and Phylogenetic Analysis

The diversity of biosynthetic gene clusters identified in these isolates suggests substantial potential for the discovery of additional bioactive metabolites through targeted cultivation and chemical ecology studies.

Future investigations should prioritise structure elucidation and purification of lead compounds, alongside toxicity evaluation in mammalian models to determine their suitability for further development as therapeutic agents. The potency of these antimicrobial agents necessitates continued evaluation of their mechanisms of action, potential for resistance development, and in vivo pharmacokinetics and safety profiles prior to consideration for clinical application.

Preliminary cytotoxicity screening conducted against human hepatocellular carcinoma cells (HepG2) and Chinese hamster ovary cells (CHO) revealed that crude extracts exhibited cytotoxic effects at concentrations of 50–100 μg/mL, corresponding to selectivity indices (SI = CC_5_₀/MIC) ranging from 4 to 20 for the most active compounds (Figure S13). These selectivity indices, while modest, indicate a therapeutic window that merits further investigation. However, the translational potential of these compounds requires careful and comprehensive evaluation of their safety profiles and selectivity.

### 4.2 Safety and Translational Potential

The antimicrobial agents produced by PAU‐7 and PAU‐15 demonstrated potent in vitro activity against clinically significant multidrug‐resistant pathogens, including methicillin‐resistant *Staphylococcus aureus* (MRSA) and carbapenem‐resistant Enterobacteriaceae (CRE), with minimum inhibitory concentrations (MICs) ranging from 2.5 to 12.5 μg/mL.

## 5 Conclusions

Nigerian agricultural soils harbour diverse Streptomyces populations with substantial biosynthetic potential for putatively novel antimicrobial compounds. The integration of genomic mining, chemical analysis, and bioactivity screening has proven effective for natural product discovery while revealing insights into factors influencing microbial secondary metabolite production in tropical soil ecosystems. The activities observed against multidrug‐resistant pathogens provide evidence for continued exploration of soil microbiomes as sources of therapeutic agents.

## Author Contributions

**David Adeiza Zakari:** conceptualization, methodology, software, writing – review and editing, writing – original draft, formal analysis, project administration. **Sarafadeen Olateju Kareem:** conceptualization, methodology, supervision, writing – review and editing. **Obuotor Tolulope Mobolaji:** methodology, writing – review and editing, formal analysis, supervision. **Akinloye Oluseyi Adeboye:** conceptualization, writing – review and editing, supervision. **Adefila Moyosore Adebimpe:** conceptualization, methodology, writing – review and editing, writing – original draft.

## Conflicts of Interest

The authors declare no conflicts of interest.

## Supporting information


**Table S1:** emi470356‐sup‐0001‐TableS1‐S3‐FigureS1‐S13.docx. ^1^H NMR and ^13^C NMR chemical shift data for characterised compounds.
**Table S2:** COSY and HSQC correlation data.
**Table S3:** HMBC long‐range correlations and chemical shift summary.
**Figure S1:** LC–MS/MS chromatogram and fragmentation pattern—Streptolide A.
**Figure S2:** LC–MS/MS chromatogram and fragmentation pattern—Griseamide B.
**Figure S3:** LC–MS/MS chromatogram and fragmentation pattern—Olivacin C.
**Figure S4:** LC–MS/MS chromatogram and fragmentation pattern—Violapeptide.
**Figure S5:** LC–MS/MS chromatogram and fragmentation pattern—Coelicoside.
**Figure S6:** LC–MS/MS chromatogram and fragmentation pattern—Nigericin X.
**Figures S7–S12:** Additional LC–MS/MS data (text representation).
**Figure S7:** Streptolide A—Expanded m/z 200–250 region. Shows characteristic macrolactone fragmentation with m/z 205 base peak. Fine structure shows isotope patterns consistent with one nitrogen atom in molecule.
**Figure S8:** Griseamide B—Expanded m/z 150–250 region. Shows cyclic peptide diagnostic ions including m/z 213 (cyclic core). Multiple ions in 140–200 range indicate non‐proteinogenic amino acid content.
**Figure S9:** Olivacin C—expanded m/z 250–450 region. Shows hybrid PKS‐NRPS characteristic fragments. Aromatic region (m/z 120–180) shows dimethoxybenzene pattern. Polyketide region (m/z 300–450) shows extended chain.
**Figure S10:** Violapeptide—expanded m/z 100–300 region. Shows complete peptide fragmentation ladder with characteristic amino acid‐derived ions. Linear structure confirmed by sequential fragmentation pattern.
**Figure S11:** Coelicoside—expanded m/z 120–350 region. Shows O‐glycoside diagnostic fragmentation. Glucose fragments clearly evident at m/z 163 and 145. Aglycone‐sugar junction fragments at m/z 307–325.
**Figure S12:** Nigericin X—expanded m/z 300–600 region. Shows polyether macrocyclic structure stability. Multiple sequential loss peaks indicating regular branching. Amide functionality retained in higher m/z fragments.
**Figure S13:** Cytotoxicity profiles of selected crude extracts and purified compounds.

## Data Availability

The datasets generated during this study are available in the GenBank repository under accession numbers OR451230–OR451252 (https://www.ncbi.nlm.nih.gov/genbank/) and in the NCBI BioProject database (Project ID: PRJNA1045823). The 16S rRNA gene sequences are accessible via NCBI under BioProject accession PRJNA1045823 and corresponding GenBank entries OR451230–OR451252. LC–MS/MS raw data have been deposited in the MassIVE repository under accession MSV000093847. All other data supporting the findings of this study are available from the corresponding author upon reasonable request.
