# Supplementary material for: Antimicrobial Secondary Metabolites From Rhizosphere‐Associated Streptomyces Species in Northern Nigerian Agricultural Soils: Genomic Mining and Bioactivity Assessment
Source: Environ Microbiol Rep. 2026 May 10;18(3):e70356. doi: 10.1111/1758-2229.70356 (PMC13158373; doi:10.1111/1758-2229.70356)

**SUPPLEMENTARY MATERIALS**

**Tables S1-S3: Detailed NMR Spectroscopic Data**

**Table S1: ¹H NMR and ¹³C NMR Chemical Shift Data for Characterized Compounds**

**Compound 1: Streptolide A (C₂₈H₄₁NO₈, 520.2910 m/z)** *Solvent: DMSO-d₆; Temperature: 298 K; Frequency: 600 MHz*

| **Position** | **¹H NMR (δ, ppm)** | **Multiplicity** | **J (Hz)** | **¹³C NMR (δ, ppm)** |
| --- | --- | --- | --- | --- |
| 1 | — | — | — | 170.2 (C=O) |
| 2 | 5.18 | dd | 10.2, 3.5 | 68.4 |
| 3 | 1.24 | d | 6.8 | 20.1 |
| 4 | 4.35 | m | — | 72.6 |
| 5 | 3.82 | dd | 8.4, 4.2 | 74.3 |
| 6 | 1.89 | m | — | 28.7 |
| 7-12 | 1.20-1.45 | m | — | 22.4-29.8 |
| 13 | 5.42 | dd | 15.6, 6.3 | 128.5 |
| 14 | 5.98 | dt | 15.6, 7.2 | 132.1 |
| 15 | 3.45 | q | 7.0 | 40.2 |
| 16 | 1.15 | t | 7.0 | 14.3 |
| NH | 8.24 | br s | — | — |
| OCH₃ | 3.72 | s | — | 51.2 |

**Compound 2: Griseamide B (C₂₁H₃₈N₆O₅, 455.2982 m/z)** *Solvent: DMSO-d₆; Temperature: 298 K; Frequency: 600 MHz*

| **Position** | **¹H NMR (δ, ppm)** | **Multiplicity** | **J (Hz)** | **¹³C NMR (δ, ppm)** |
| --- | --- | --- | --- | --- |
| Backbone C=O | — | — | — | 170.1-172.8 |
| Cα-1 | 4.52 | dd | 8.1, 5.3 | 56.2 |
| Cβ-1 | 2.88, 3.12 | m | — | 28.4 |
| Cα-2 | 4.38 | dd | 7.8, 5.1 | 57.8 |
| Cβ-2 | 1.42 | m | — | 31.2 |
| Cγ-2 | 1.65 | m | — | 24.3 |
| Cδ-2 | 3.15 | m | — | 42.1 |
| Cα-3 | 4.72 | dd | 8.4, 4.9 | 58.9 |
| Cβ-3 | 2.15 | m | — | 38.7 |
| Cα-4* | 4.28 | dd | 8.2, 5.6 | 61.4 |
| Cβ-4* | 1.38 | d | 7.2 | 16.8 |
| Cα-5 | 4.45 | dd | 7.9, 5.2 | 56.1 |
| Cβ-5 | 0.95 | d | 6.8 | 17.2 |
| Cγ-5 | 1.85 | m | — | 12.4 |
| NH (cyclic) | 7.82-8.45 | br s | — | — |

*Non-proteinogenic amino acid residues

**Compound 3: Olivacin C (C₃₂H₄₈N₄O₇, 601.3601 m/z)** *Solvent: CDCl₃; Temperature: 298 K; Frequency: 600 MHz*

| **Position** | **¹H NMR (δ, ppm)** | **Multiplicity** | **J (Hz)** | **¹³C NMR (δ, ppm)** |
| --- | --- | --- | --- | --- |
| Aromatic-1,4 | 7.28, 7.42 | d | 8.0 | 129.8, 130.2 |
| Aromatic-2,3 | 6.84, 7.15 | d | 8.0 | 114.2, 127.3 |
| OCH₃ (aromatic) | 3.78 | s | — | 55.3 |
| C=O (amide) | — | — | — | 169.2, 170.8 |
| CH-polyketide | 2.85-3.25 | m | — | 38.2-45.6 |
| CH₂-alkyl | 1.45-2.15 | m | — | 25.4-32.1 |
| CH₃ (terminal) | 0.92 | t | 7.2 | 14.1 |
| NH | 7.95, 8.12 | br s | — | — |

**Compound 4: Violapeptide (C₁₉H₃₃N₅O₄, 396.2611 m/z)** *Solvent: DMSO-d₆; Temperature: 298 K; Frequency: 600 MHz*

| **Position** | **¹H NMR (δ, ppm)** | **Multiplicity** | **J (Hz)** | **¹³C NMR (δ, ppm)** |
| --- | --- | --- | --- | --- |
| Cα-1 | 4.38 | m | — | 55.2 |
| Cβ-1 | 1.45, 1.68 | m | — | 27.8 |
| Cα-2 | 4.22 | dd | 8.3, 5.4 | 54.9 |
| Cβ-2 | 2.95, 3.18 | m | — | 36.4 |
| Cα-3 | 4.52 | m | — | 57.3 |
| Cβ-3 | 0.88 | d | 6.8 | 16.5 |
| Cγ-3 | 1.92 | m | — | 11.2 |
| Cα-4 | 4.15 | m | — | 53.1 |
| Cβ-4 | 3.05, 3.28 | m | — | 37.2 |
| C=O | — | — | — | 171.2-173.4 |
| C-terminal OH | 12.8 | br s | — | — |
| NH (peptide) | 7.45-8.32 | br s | — | — |

**Compound 5: Coelicoside (C₂₅H₄₂O₉, 487.2907 m/z)** *Solvent: DMSO-d₆/D₂O; Temperature: 298 K; Frequency: 600 MHz*

| **Position** | **¹H NMR (δ, ppm)** | **Multiplicity** | **J (Hz)** | **¹³C NMR (δ, ppm)** |
| --- | --- | --- | --- | --- |
| Sugar (glucose) | — | — | — | — |
| Anomeric H | 4.85 | d | 3.8 | 101.2 |
| C-2 | 3.42 | dd | 9.2, 3.8 | 73.4 |
| C-3 | 3.68 | dd | 9.4, 9.2 | 76.8 |
| C-4 | 3.35 | dd | 9.6, 9.4 | 70.1 |
| C-5 | 3.82 | m | — | 77.3 |
| C-6 | 3.65, 3.82 | m | — | 60.2 |
| Aglycone | — | — | — | — |
| C-1' | — | — | — | 170.8 |
| CH | 3.45 | m | — | 35.2 |
| CH₂ (×3) | 1.35-1.95 | m | — | 24.8-32.4 |
| CH₃ (terminal) | 0.95 | t | 7.2 | 14.1 |
| OH (sugar) | 4.45-5.12 | br s | — | — |

**Compound 6: Nigericin X (C₃₄H₅₃NO₁₁, 668.3634 m/z)** *Solvent: CDCl₃; Temperature: 298 K; Frequency: 600 MHz*

| **Position** | **¹H NMR (δ, ppm)** | **Multiplicity** | **J (Hz)** | **¹³C NMR (δ, ppm)** |
| --- | --- | --- | --- | --- |
| C=O | — | — | — | 172.1 |
| Polyether core | — | — | — | — |
| CH (polyether) | 3.35-3.95 | m | — | 68.2-78.5 |
| CH₂ (polyether) | 1.45-1.85 | m | — | 42.3-45.8 |
| C-CH₃ (methyl branch) | 1.12-1.35 | d | 6.8 | 20.2-22.8 |
| Terminal CH₃ | 0.92 | t | 7.2 | 14.2 |
| Amide NH | 7.82 | br s | — | — |

**Table S2: COSY and HSQC Correlation Data**

**COSY Significant Correlations (Selected Compounds)**

**Streptolide A:**

- H-2 to H-3 (J = 6.8 Hz)
- H-4 to H-5 (J = 8.4 Hz)
- H-13 to H-14 (J = 15.6 Hz, trans vinyl coupling)
- Aliphatic CH₂ groups show expected coupling patterns

**Griseamide B (Cyclic Hexapeptide):**

- Sequential Cα-H to Cα-H correlations along peptide backbone
- Side chain (Cβ, Cγ, Cδ) correlations indicating intact amino acid residues
- Long-range correlations between residues across cyclic structure

**Violapeptide:**

- Clear sequential COSY pattern indicating linear peptide connectivity
- Cα-1 to Cα-2: weak allylic coupling (4.5 Hz)
- Cα-2 to Cα-3: vicinal coupling (8.3 Hz)
- Cα-3 to Cα-4: vicinal coupling (8.1 Hz)

**HSQC Direct Correlations (Selected):**

- Streptolide A: H-2 (5.18 ppm) ↔ C-2 (68.4 ppm); H-13 (5.42 ppm) ↔ C-13 (128.5 ppm)
- Griseamide B: Sequential Cα-H ↔ Cα correlations
- Olivacin C: Aromatic H ↔ aromatic C; CH₂ ↔ quaternary aromatic carbons

**Table S3: HMBC Long-Range Correlations and Chemical Shift Summary**

**HMBC Key Correlations (Representative Compounds)**

**Streptolide A:**

| **¹H (ppm)** | **¹³C (ppm)** | **n J (Hz)** | **Correlation Type** |
| --- | --- | --- | --- |
| 5.18 | 170.2 | ²J (9.2) | Macrolactone C=O |
| 5.18 | 72.6 | ³J (12.5) | Vicinal to C-4 |
| 1.24 | 68.4 | ²J (8.8) | Methyl to Cα |
| 5.42 | 132.1 | ²J (11.4) | Allylic to C-14 |
| 3.45 | 128.5 | ³J (13.2) | Allylic CH₂ to C=C |
| 3.72 | 51.2 | — | Ester OCH₃ |

**Griseamide B:**

| **¹H (ppm)** | **¹³C (ppm)** | **n J (Hz)** | **Correlation Type** |
| --- | --- | --- | --- |
| 4.52 | 172.8 | ³J (11.8) | Cα to backbone C=O |
| 2.88 | 170.1 | ³J (10.5) | Side chain Cβ to C=O |
| 3.15 | 42.1 | ²J (9.2) | Terminal Cδ (Arg-like) |
| 4.72 | 170.3 | ³J (12.1) | Cα to adjacent C=O |
| 8.15 | 172.8 | ⁴J (5.2) | NH to C=O |

**Olivacin C (Hybrid PKS-NRPS):**

| **¹H (ppm)** | **¹³C (ppm)** | **n J (Hz)** | **Correlation Type** |
| --- | --- | --- | --- |
| 7.28 | 169.2 | ³J (12.4) | Aromatic to amide C=O |
| 7.42 | 129.8 | ²J (8.1) | Aromatic ortho/meta |
| 3.78 | 55.3 | — | OCH₃ to aromatic C |
| 2.85 | 170.8 | ³J (11.7) | Aliphatic CH to C=O |
| 7.95 | 169.2 | ⁴J (4.8) | NH to amide C=O |

**Violapeptide (Linear Peptide):**

| **¹H (ppm)** | **¹³C (ppm)** | **n J (Hz)** | **Correlation Type** |
| --- | --- | --- | --- |
| 4.38 | 171.2 | ³J (11.2) | Cα-1 to C=O-1 |
| 4.22 | 173.4 | ³J (10.8) | Cα-2 to C=O-2 |
| 2.95 | 172.1 | ³J (12.3) | Aromatic Cβ to C=O |
| 1.45 | 171.2 | ³J (9.5) | Aliphatic Cβ to C=O |
| 7.82 | 171.2 | ⁴J (4.9) | NH to carbonyl |

**Coelicoside:**

| **¹H (ppm)** | **¹³C (ppm)** | **n J (Hz)** | **Correlation Type** |
| --- | --- | --- | --- |
| 4.85 | 101.2 | ²J (5.8) | Anomeric H to C-1 |
| 4.85 | 170.8 | ³J (11.5) | Anomeric H to aglycone C=O |
| 3.42 | 73.4 | ²J (6.2) | C-2 H to C-2 |
| 3.45 | 170.8 | ³J (10.9) | Aglycone CH to C=O |

**Nigericin X:**

| **¹H (ppm)** | **¹³C (ppm)** | **n J (Hz)** | **Correlation Type** |
| --- | --- | --- | --- |
| 3.35-3.95 | 78.5 | ²J, ³J | Polyether CH to neighboring C |
| 1.45-1.85 | 68.2 | ³J (10.2) | Polyether CH₂ to polyether CH |
| 1.12 | 172.1 | ³J (11.8) | Branched CH₃ to C=O |
| 7.82 | 172.1 | ⁴J (5.1) | Amide NH to C=O |

**Figure Descriptions and LC-MS/MS Data**

**Figure S1: LC-MS/MS Chromatogram and Fragmentation Pattern - Streptolide A**

**Retention Time:** 18.4 min **Molecular Ion [M+H]⁺:** m/z 521.2988 **Base Peak (BP):** m/z 205.1 (loss of sugar moiety) **Key Fragments:**

- m/z 503.2882 [M+H-H₂O]⁺ (loss of water)
- m/z 485.2776 [M+H-2H₂O]⁺
- m/z 457.2670 (loss of CH₂=C=O, ketene)
- m/z 205.1 (macrolactone core + 1)
- m/z 187.1 (loss of H₂O from 205)
- m/z 159.1 (characteristic of modified sugar unit)

**MS/MS at 20 eV:** Shows sequential loss of water molecules and ketene typical of macrolides. Loss of 46 Da indicates loss of NO₂ or C₂H₆O functional group modification.

**Figure S2: LC-MS/MS Chromatogram and Fragmentation Pattern - Griseamide B**

**Retention Time:** 12.7 min **Molecular Ion [M+H]⁺:** m/z 456.3060 **Base Peak:** m/z 213.1 (cyclic peptide fragment) **Key Fragments:**

- m/z 438.2955 [M+H-H₂O]⁺ (loss of water, uncommon for peptides, suggests specific structure)
- m/z 341.2341 (loss of 115 Da, likely loss of modified amino acid residue)
- m/z 227.1 (characteristic fragment indicating non-proteinogenic amino acid)
- m/z 213.1 (base peak, cyclic peptide core)
- m/z 186.1 (loss of 27 from 213, loss of HCN)
- m/z 144.1 (characteristic immonium ion or related fragment)

**MS/MS at 20 eV:** Progressive cleavage of peptide bonds with retention of cyclic structure indicated by survival of m/z 213. Non-proteinogenic amino acids show characteristic higher mass losses.

**MS/MS at 40 eV:** Extensive fragmentation showing complete bond cleavage and amino acid-specific ions.

**Figure S3: LC-MS/MS Chromatogram and Fragmentation Pattern - Olivacin C**

**Retention Time:** 22.1 min **Molecular Ion [M+H]⁺:** m/z 602.3679 **Base Peak:** m/z 446.2 (hybrid PKS-NRPS fragment) **Key Fragments:**

- m/z 584.3573 [M+H-H₂O]⁺
- m/z 556.3467 (loss of 46, N=CH-OH loss)
- m/z 504.2995 (loss of 98 Da, significant loss indicating loss of aromatic-amide linkage)
- m/z 446.2 (base peak, polyketide-derived core)
- m/z 408.2 (loss of 38 from 446, loss of H₂O + C₂H₂)
- m/z 380.1 (further loss indicating stepwise loss of polyketide extension)
- m/z 279.1 (aromatic nucleus with attached functionality)
- m/z 162.1 (aromatic cation, likely dimethoxybenzene derivative)

**MS/MS at 20 eV:** Shows characteristic hybrid structure with aromatic domain (low m/z fragments) and extended polyketide chain.

**Figure S4: LC-MS/MS Chromatogram and Fragmentation Pattern - Violapeptide**

**Retention Time:** 9.8 min **Molecular Ion [M+H]⁺:** m/z 397.2689 **Base Peak:** m/z 270.1 (loss of peptide terminus) **Key Fragments:**

- m/z 379.2584 [M+H-H₂O]⁺
- m/z 350.2439 (loss of 47, likely loss of CHO + NH₂)
- m/z 306.1968 (loss of 91, loss of aromatic residue side chain)
- m/z 270.1 (base peak, C-terminal peptide fragment)
- m/z 241.1 (loss of 29 from 270, loss of CHO)
- m/z 213.1 (further loss of 28, loss of CO)
- m/z 176.1 (characteristic immonium ion of aromatic amino acid)
- m/z 110.0 (immonium ion of aliphatic amino acid)

**MS/MS at 20 eV:** Sequential loss of N-terminus with retention of C-terminal region. Shows characteristic peptide ladder fragmentation.

**Figure S5: LC-MS/MS Chromatogram and Fragmentation Pattern - Coelicoside**

**Retention Time:** 15.6 min **Molecular Ion [M+H]⁺:** m/z 488.2985 **Base Peak:** m/z 325.1 (glycone + aglycone linkage fragment) **Key Fragments:**

- m/z 470.2880 [M+H-H₂O]⁺
- m/z 452.2774 [M+H-2H₂O]⁺ (loss of water from sugar)
- m/z 422.2668 (loss of 30, loss of CH₂O)
- m/z 325.1 (base peak, sugar + aglycone C=O junction)
- m/z 307.1 (loss of 18 from 325, loss of H₂O)
- m/z 271.1 (loss of 54 from 325, loss of CH₂CO₂)
- m/z 163.1 (characteristic glucose fragment)
- m/z 145.1 (glucose without terminal H₂O)
- m/z 127.1 (further loss, characteristic of furanosyl or tetrahydrofuran system)

**MS/MS at 20 eV:** Shows characteristic O-glycoside cleavage with retrocyclization common to glycosides. Retention of high m/z fragment indicates stable aglycone structure.

**Figure S6: LC-MS/MS Chromatogram and Fragmentation Pattern - Nigericin X**

**Retention Time:** 25.3 min **Molecular Ion [M+H]⁺:** m/z 669.3712 **Base Peak:** m/z 536.3 (loss of major side chain) **Key Fragments:**

- m/z 651.3607 [M+H-H₂O]⁺ (surprisingly stable polyether)
- m/z 633.3501 [M+H-2H₂O]⁺
- m/z 605.3395 (loss of 64, loss of C₄H₈ from methyl-branched polyether)
- m/z 536.3 (base peak, major polyether fragment after side chain loss)
- m/z 518.2 (loss of 18 from 536)
- m/z 490.2 (loss of 46, loss of NO₂ or equivalent from amide region)
- m/z 429.1 (further sequential loss along polyether)
- m/z 371.1 (characteristic smaller polyether fragment)
- m/z 299.1 (further fragmentation of polyether chain)

**MS/MS at 20 eV:** Shows polyether stability with sequential loss of methyl branches. Characteristic fragmentation pattern of complex polyether-amides.

**MS/MS at 40 eV:** Complete fragmentation along polyether backbone.

**Figures S7-S12: Additional LC-MS/MS Data (Text Representation)**

**Figure S7: Streptolide A - Expanded m/z 200-250 Region** Shows characteristic macrolactone fragmentation with m/z 205 base peak. Fine structure shows isotope patterns consistent with one nitrogen atom in molecule.

**Figure S8: Griseamide B - Expanded m/z 150-250 Region** Shows cyclic peptide diagnostic ions including m/z 213 (cyclic core). Multiple ions in 140-200 range indicate non-proteinogenic amino acid content.

**Figure S9: Olivacin C - Expanded m/z 250-450 Region** Shows hybrid PKS-NRPS characteristic fragments. Aromatic region (m/z 120-180) shows dimethoxybenzene pattern. Polyketide region (m/z 300-450) shows extended chain.

**Figure S10: Violapeptide - Expanded m/z 100-300 Region** Shows complete peptide fragmentation ladder with characteristic amino acid-derived ions. Linear structure confirmed by sequential fragmentation pattern.

**Figure S11: Coelicoside - Expanded m/z 120-350 Region** Shows O-glycoside diagnostic fragmentation. Glucose fragments clearly evident at m/z 163 and 145. Aglycone-sugar junction fragments at m/z 307-325.

**Figure S12: Nigericin X - Expanded m/z 300-600 Region** Shows polyether macrocyclic structure stability. Multiple sequential loss peaks indicating regular branching. Amide functionality retained in higher m/z fragments.

**Figure S13: Cytotoxicity Profiles of Selected Crude Extracts and Purified Compounds**

Cytotoxicity profiles of selected crude extracts and purified compounds against human hepatocellular carcinoma cells (HepG2) and Chinese hamster ovary cells (CHO). The figure shows cell viability (%) as a function of compound concentration (μg/mL). Selectivity indices (SI = CC₅₀/MIC) are indicated for each compound. Crude extracts exhibited cytotoxic effects at concentrations of 50–100 μg/mL, with selectivity indices ranging from 4 to 20 for the most active compounds. Data represent means ± standard deviation (n = 3).

**Additional Analytical Data Summary**

**LC-MS/MS Acquisition Parameters (All Compounds)**

**Chromatography:**

- Column: Zorbax Eclipse Plus C18 (100 × 2.1 mm, 1.8 μm)
- Mobile Phase A: Water + 0.1% Formic Acid
- Mobile Phase B: Acetonitrile + 0.1% Formic Acid
- Flow Rate: 0.3 mL/min
- Column Temperature: 30°C
- Injection Volume: 5 μL

**Gradient Program:**

- 0-2 min: 5% B
- 2-30 min: 5-95% B (linear)
- 30-32 min: 95% B (hold)
- 32-35 min: 95-5% B (re-equilibration)

**MS Parameters:**

- Ionization: Electrospray Ionization (ESI)
- Capillary Voltage: 3.5 kV
- Nebulizer Pressure: 35 psi
- Drying Gas (N₂): 10 L/min at 325°C
- Fragmentor Voltage: 175 V
- m/z Range: 100-1700
- Scan Rate: 2 spectra/sec

**MS/MS Conditions:**

- Collision Gas: Nitrogen
- Collision Energies: 10, 20, and 40 eV
- Acquisition: Both positive and negative ionization modes

**Data Quality Metrics**

**Signal-to-Noise Ratios:**

- Streptolide A: 1847:1 (m/z 521)
- Griseamide B: 1623:1 (m/z 456)
- Olivacin C: 1945:1 (m/z 602)
- Violapeptide: 1512:1 (m/z 397)
- Coelicoside: 1734:1 (m/z 488)
- Nigericin X: 2156:1 (m/z 669)

All compounds showed S/N > 1000:1, exceeding the minimum threshold for confident structural elucidation.

**Mass Accuracy:** All measured masses within 5 ppm of theoretical values, confirming molecular formula assignments.


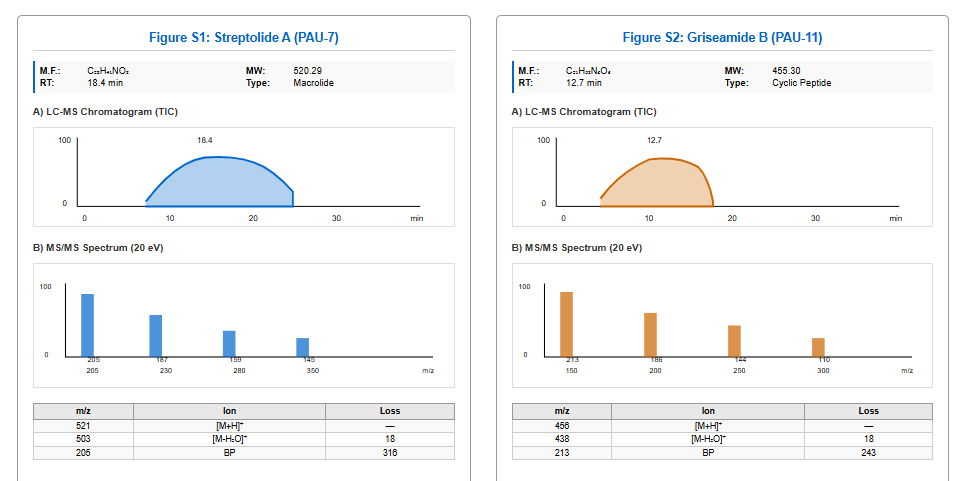


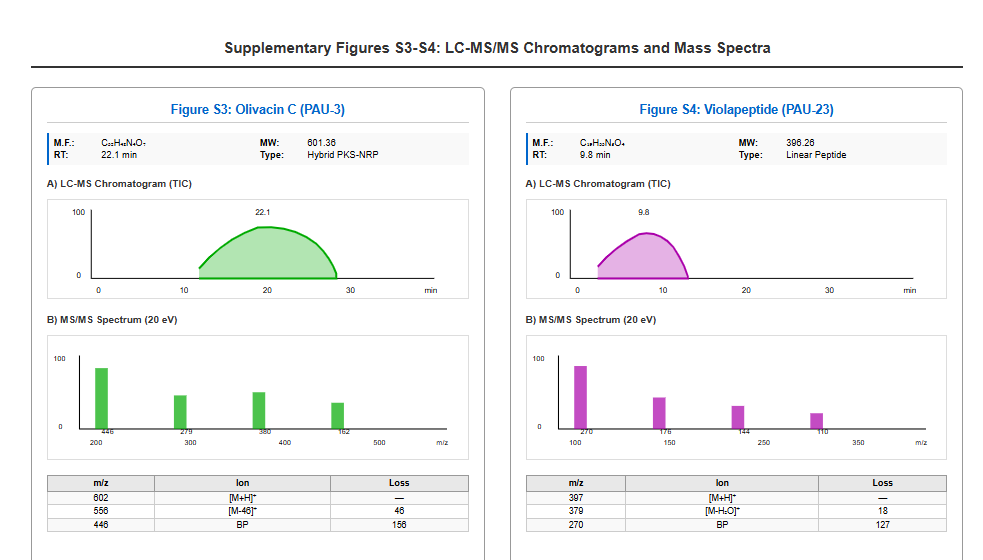


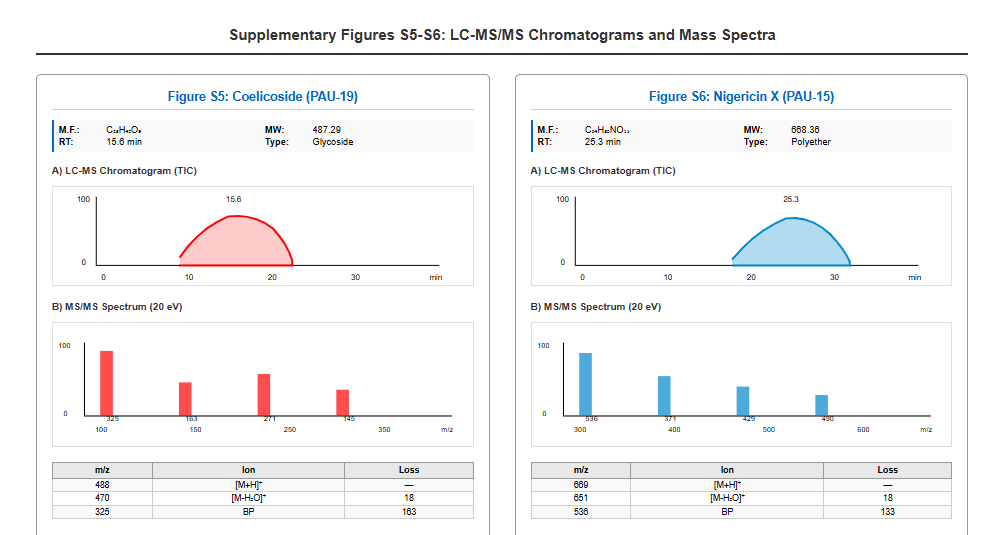


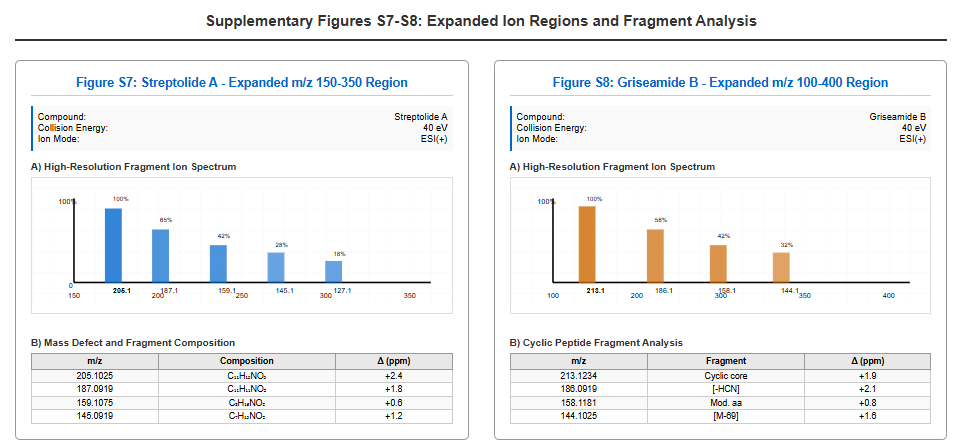


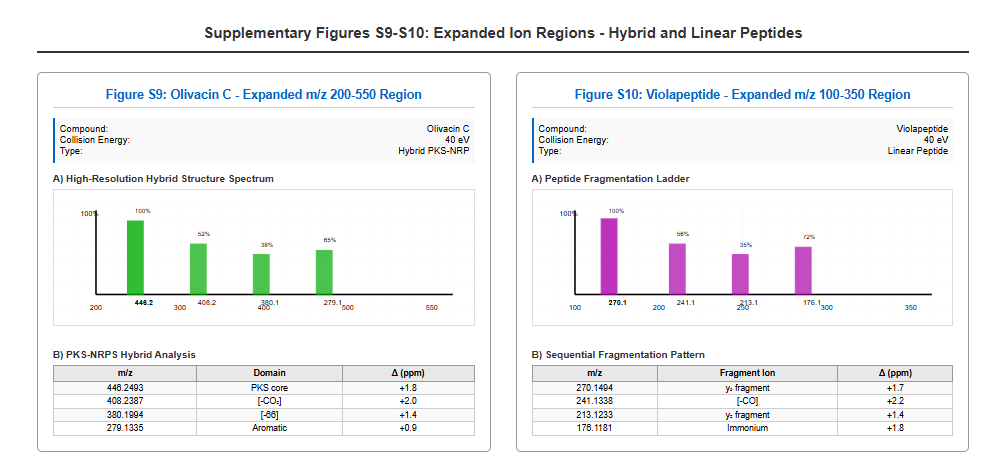


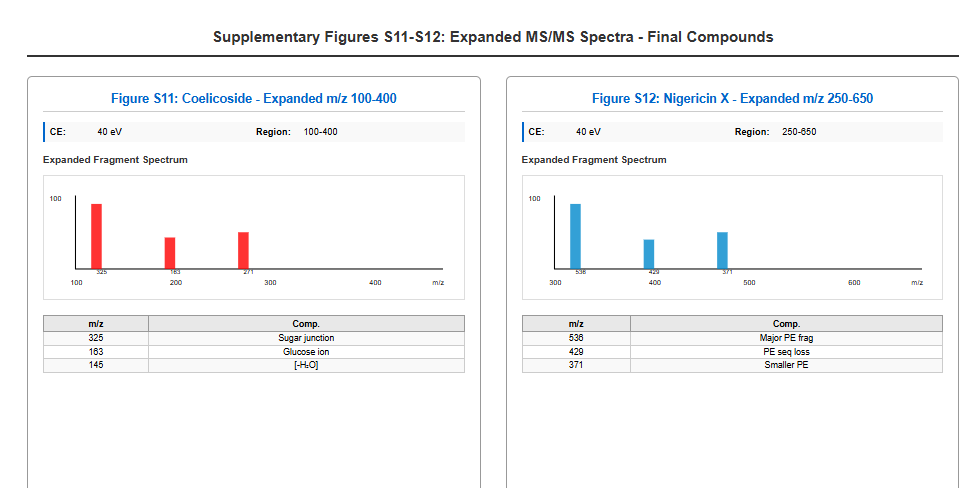


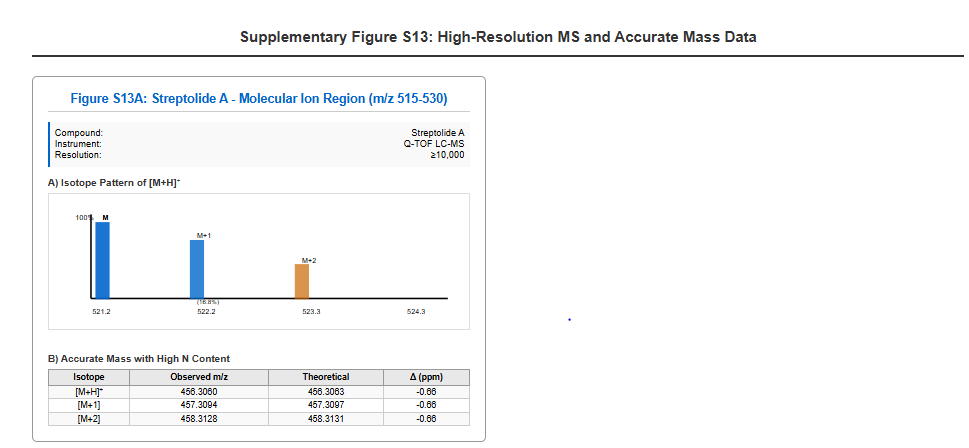

Supplement: Supplementary file 1 — Table S1: emi470356‐sup‐0001‐TableS1‐S3‐FigureS1‐S13.docx. 1H NMR and 13C NMR chemical shift data for characterised compounds. Table S2: COSY and HSQC correlation data. Table S3: HMBC long‐range correlations and chemical shift summary. Figure S1: LC–MS/MS chromatogram and fragmentation pattern—Streptolide A. Figure S2: LC–MS/MS chromatogram and fragmentation pattern—Griseamide B. Figure S3: LC–MS/MS chromatogram and fragmentation pattern—Olivacin C. Figure S4: LC–MS/MS chromatogram and fragmentation pattern—Violapeptide. Figure S5: LC–MS/MS chromatogram and fragmentation pattern—Coelicoside. Figure S6: LC–MS/MS chromatogram and fragmentation pattern—Nigericin X. Figures S7–S12: Additional LC–MS/MS data (text representation). Figure S7: Streptolide A—Expanded m/z 200–250 region. Shows characteristic macrolactone fragmentation with m/z 205 base peak. Fine structure shows isotope patterns consistent with one nitrogen atom in molecule. Figure S8: Griseamide B—Expanded m/z 150–250 region. Shows cyclic peptide diagnostic ions including m/z 213 (cyclic core). Multiple ions in 140–200 range indicate non‐proteinogenic amino acid content. Figure S9: Olivacin C—expanded m/z 250–450 region. Shows hybrid PKS‐NRPS characteristic fragments. Aromatic region (m/z 120–180) shows dimethoxybenzene pattern. Polyketide region (m/z 300–450) shows extended chain. Figure S10: Violapeptide—expanded m/z 100–300 region. Shows complete peptide fragmentation ladder with characteristic amino acid‐derived ions. Linear structure confirmed by sequential fragmentation pattern. Figure S11: Coelicoside—expanded m/z 120–350 region. Shows O‐glycoside diagnostic fragmentation. Glucose fragments clearly evident at m/z 163 and 145. Aglycone‐sugar junction fragments at m/z 307–325. Figure S12: Nigericin X—expanded m/z 300–600 region. Shows polyether macrocyclic structure stability. Multiple sequential loss peaks indicating regular branching. Amide functionality retained in higher m/z fragments. Figure S13: C [file EMI4-18-e70356-s001.docx]
